# Fluoxetine Induces Proliferation and Inhibits Differentiation of Hypothalamic Neuroprogenitor Cells *In Vitro*

**DOI:** 10.1371/journal.pone.0088917

**Published:** 2014-03-05

**Authors:** Lígia Sousa-Ferreira, Célia Aveleira, Mariana Botelho, Ana Rita Álvaro, Luís Pereira de Almeida, Cláudia Cavadas

**Affiliations:** 1 CNC - Center for Neuroscience and Cell Biology, University of Coimbra, Coimbra, Portugal; 2 Department of Biology and Environment University of Trás-os-Montes and Alto Douro, Vila Real, Portugal; 3 Faculty of Pharmacy, University of Coimbra, Coimbra, Portugal; CRCHUM-Montreal Diabetes Research Center, Canada

## Abstract

A significant number of children undergo maternal exposure to antidepressants and they often present low birth weight. Therefore, it is important to understand how selective serotonin reuptake inhibitors (SSRIs) affect the development of the hypothalamus, the key center for metabolism regulation. In this study we investigated the proliferative actions of fluoxetine in fetal hypothalamic neuroprogenitor cells and demonstrate that fluoxetine induces the proliferation of these cells, as shown by increased neurospheres size and number of proliferative cells (Ki-67^+^ cells). Moreover, fluoxetine inhibits the differentiation of hypothalamic neuroprogenitor cells, as demonstrated by decreased number of mature neurons (Neu-N^+^ cells) and increased number of undifferentiated cells (SOX-2^+^ cells). Additionally, fluoxetine-induced proliferation and maintenance of hypothalamic neuroprogenitor cells leads to changes in the mRNA levels of appetite regulator neuropeptides, including Neuropeptide Y (NPY) and Cocaine-and-Amphetamine-Regulated-Transcript (CART). This study provides the first evidence that SSRIs affect the development of hypothalamic neuroprogenitor cells *in vitro* with consequent alterations on appetite neuropeptides.

## Introduction

Food intake and body weight are centrally regulated by the hypothalamus, where arcuate nucleus (ARC) neurons sense and integrate peripheral signals of nutrition to downstream circuits [1], [2]. ARC neurons are divided in two populations acting together to regulate appetite: the orexigenic NPY/AgRP (Neuropeptide Y/Agouti-Related Protein) neurons and the anorexigenic POMC/CART (Pro-OpioMelanocortin/Cocaine-and-Amphetamine-Regulated-Transcript) neurons.

Adult hypothalamic neurogenesis occurs at low rates in rodents but it is essential for body weight and feeding regulation [3], [4]. During the embryonic period, hypothalamic neuronal precursors are generated between days E10.5 and E14.5 [5], [6] and persist until adulthood [7]. In accordance, neuroprogenitor cells can be isolated from fetal and adult hypothalamus and these cells express neuropeptides important for the regulation of feeding [8].

Proliferation of hypothalamic neuroprogenitor cells during the perinatal period is influenced by maternal nutrition and hormones availability in mice [9], [10]. Notably, this will result in body weight and appetite defects in newborns that persist after weaning [11]. Moreover, these feeding alterations indicate a possible change of neuropeptides levels in hypothalamic cells. Based on these studies we can hypothesize that any molecule affecting hypothalamic cell proliferation during the neurodevelopment period can modify the programming of hypothalamic satiety pathways leading to persistent changes in newborns homeostasis.

Selective serotonin reuptake inhibitors (SSRIs) are antidepressant drugs also known for their neurogenic effect in perinatal hippocampal and cerebellar neuroprogenitor cells [12], [13]. Additionally, SSRIs obtained from maternal lactation have proven to restore hippocampal neurogenesis in stressed rat offspring [14]. Nevertheless, the potential proliferative effect of SSRI in the perinatal hypothalamus is unknown and requires investigation since SSRIs are the drug of choice for treating depressed pregnant and postpartum women [15]. In fact, there is a 10–16% prevalence of depression during pregnancy and 25% of depressed women continue antidepressant use during pregnancy [16], [17]. As most SSRIs reach the fetus via the placenta and are detectable in breast milk [18], [19] a significant number of children are exposed to SSRIs during critical phases of hypothalamic neurodevelopment. Accordingly, it has been reported that maternal exposure to SSRIs results in low birth weight and modifications of the hypothalamic-pituitary-adrenal axis of human and rodent newborns [20], [21], [22], [23].

Therefore, in this study we investigated whether the SSRI fluoxetine alters the proliferation and differentiation of rat embryonic hypothalamic neuroprogenitor cells. Moreover, using an *in vitro* model previously described by our group [8], we evaluated the effect of fluoxetine in the expression levels of hypothalamic neuropeptides that regulate food intake, including orexigenic (NPY and AgRP) and anorexigenic (POMC and CART) neuropeptides.

## Materials and Methods

### Ethics statement

All experimental procedures were performed in accordance with the European Union Directive 86/609/EEC for the care and use of laboratory animals. In addition, animals were housed in a licensed animal facility (international Animal Welfare Assurance number 520.000.000.2006) and the CNC animal experimentation board approved the utilization of animals for this project. Moreover, people working with animals have received appropriate education (FELASA course) as required by the Portuguese authorities.

### Embryonic hypothalamic neurospheres culture

Hypothalamic neuroprogenitor cells were isolated and cultured as floating neurospheres as previously described [8]. Briefly, hypothalamic tissue dissected from rat embryos (E18-19) were mechanically dissociated by means of a Pasteur pipette. Cells were suspended in DMEM-F12/Glutamax supplemented with growth factors (10 ng/ml fibroblast growth factor-2 and 10 ng/ml epidermal growth factor), 100 U/ml penicillin, 100 µg/ml streptomycin and 1% B27 supplement (all from Gibco). Every seven days of culture, neurospheres were dissociated through a P200 pipette and re-suspended in fresh DMEM-F12/Glutamax medium with growth factors, corresponding to one passage (P).

### Differentiation of hypothalamic neuroprogenitor cells

After 6–7 days in culture, neurospheres were dissociated and plated in Poly-D-Lysine coated culture plates as previously described [8]. To obtain differentiated hypothalamic neural cultures, the plated neurospheres were allowed to differentiate for 18 days in Neurobasal medium with 500 µM L-Glutamine, 2% B27 supplement, 100 U/ml penicillin and 100 µg/ml streptomycin (all from Gibco), with no growth factors. After 18 days in culture, differentiated hypothalamic neural cultures were used for immunocytochemistry or RNA extraction.

### Materials

Fluoxetine, Cytosine Arabinoside (AraC) and tyrosine kinases (Trk) receptor inhibitor K252a were purchased from Sigma-Aldrich.

### Drug treatments

A recent rodent study showed that about 80% of plasma fluoxetine is transferred to the pup after maternal injection, with high levels of fluoxetine being detected in the pup brain [24]. For this study, hypothalamic neuroprogenitor cells were incubated with fluoxetine 1 µM (Sigma); this corresponds to the plasma concentration of fluoxetine in rat pups after maternal exposure to fluoxetine (12 mg/kg) [24]. Additionally, it was described that 1 µM fluoxetine leads to a maximal increase in proliferation hippocampal and cerebellar neural stem cells cultures [12], [13].

Hypothalamic neurospheres were cultured in the presence of fluoxetine (1 µM); treatment with fluoxetine started 3 days after isolation of hypothalamic neuroprogenitor cells. Control hypothalamic neurospheres were cultured in the absence of fluoxetine.

To investigate the proliferative effects of fluoxetine and contribution of neurotrophins to the fluoxetine-induced proliferation, control and fluoxetine P3 hypothalamic neurospheres were incubated with the proliferation inhibitor AraC (10 µM) or the Trk receptor inhibitor K252a (200 nM) during the last 24 hours before RNA collection or plating.

Hypothalamic neuroprogenitor cultures were allowed to differentiate in the presence of fluoxetine (1 µM). Treatment with fluoxetine started 2 days after plating of hypothalamic neurospheres. Incubation with Trk receptor inhibitor K252a (200 nM) occurred during the last 24 hours before RNA collection or fixation. Control hypothalamic neurospheres differentiated in the absence of fluoxetine.

### Immunocytochemistry of hypothalamic neurospheres

Twenty-four hours after incubation with the inhibitors, P3 hypothalamic neurospheres were plated in Poly-D-Lysine coated 12-well culture plates and kept in culture for 1 day with fresh medium (DMEM-F12/Glutamax supplemented with growth factors) before fixation.

P3 hypothalamic neurospheres and differentiated hypothalamic neuroprogenitor cultures were fixed with 4% paraformaldehyde (Sigma) and permeabilized with 1% Triton X-100 (Sigma) followed by one hour blocking with 3% BSA (Sigma) and incubation with the primary antibody overnight at 4°C. Thereafter, the incubation with the secondary antibody was performed for one hour at room temperature. Nuclei were stained with 4′6-diamidino-2-phenylindoline, DAPI (1∶5000, Applichem).The primary antibodies used were: mouse anti-Ki-67 (1∶50; NovoCastra), mouse anti-Neu-N (1∶500, Chemicon) and mouse anti-SOX-2 (1∶200, R&D Systems). The secondary antibodies used were: goat anti-mouse Alexa Fluor 594 and goat anti-mouse Alexa Fluor 488 (1∶200, Invitrogen).

### Quantification of cell proliferation and differentiation by immunocytochemistry

To investigate the effect of fluoxetine in the proliferation and differentiation of hypothalamic neuroprogenitor cells, the percentage of Ki-67, Neu-N and SOX-2 positive cells was calculated in P3 hypothalamic neurospheres and differentiated hypothalamic neuroprogenitor cultures. Fluorescence images were recorded in the monolayer formed at the neurospheres periphery (Ki-67) or in the center of the neurospheres (Neu-N and SOX-2) using a confocal microscope (LSM 510 Meta; Zeiss). The percentage of Ki-67, Neu-N and SOX-2 positive cells was calculated in ten independent microscopic fields and normalized to total number of nuclei with DAPI staining (approximately 80–120 nuclei per microscopic field). Data are shown as percentage of control. The procedure was performed for four independent culture preparations.

### Hypothalamic neurospheres size evaluation

The size of control and fluoxetine-treated neurospheres was evaluated immediately before the next passage, using the Axiovision software (Zeiss) to determine their diameter. The neurospheres diameters oscillated between 50 and 250 µm and this interval was divided into four subintervals (<90 µm, 90–150 µm, 150–210 µm, >210 µm), to obtain the size distribution of hypothalamic neurospheres. Size distribution was determined as percentage of total number of neurospheres evaluated for each treatment. The procedure was performed for four independent culture preparations.

### Isolation of total RNA from hypothalamic neurospheres and cDNA synthesis

Hypothalamic neurospheres were collected by centrifugation and immediately frozen in dry ice as described before [8]. Samples were kept at −80°C until RNA extraction. Total RNA was extracted from P0 (1 week), P1 (2 weeks), P2 (3 weeks), P3 (4 weeks) and P4 (5 weeks) hypothalamic neurospheres, and differentiated hypothalamic neuroprogenitor cultures using the RNeasy Mini Kit (QIAGEN) according to the manufacturer's protocol for animal cells. Total amount of RNA was quantified by optical density (OD) measurements using a ND-1000 Nanodrop Spectrophotometer (Thermo Scientific) and the purity was evaluated by measuring the ratio of OD at 260 and 280 nm. In addition, RNA quality was assessed by gel electrophoresis. Prior to cDNA conversion, samples were treated with RNase-free DNAse (QIAGEN) to eliminate any contamination with genomic DNA. cDNA was obtained from the conversion of 1 µg of total RNA using the iScript cDNA Synthesis Kit (Bio-Rad) according to the manufacturer's instructions and stored at −20°C.

### PCR for evaluation of serotonergic markers

Each PCR reaction contained 50 ng of template cDNA, 0.2 µl of Phusion Polymerase (Finnzymes), 200 µM dNTPs (ThermoScientific) and 0.5 µM of reverse and forward primers, to a final volume of 20 µl. Primers sequences for tryptophan hidroxylase type 2 (TPH2), serotonin receptor type 5-HT_1_A and serotonin pre-synaptic transporter (SERT) were previously described by others [25], [26]. PCR was performed in a thermal cycler and the thermal conditions were as follow: 98°C for 30 sec; 35 cycles of 98°C for 10 sec, 64°C for 30 sec and 72°C for 15 sec; and 72°C for 10 min. Total RNA extracted from adult rat brain was used as positive control. PCR runs with RNA samples (no RT samples) and with no polymerase served as negative controls. PCR products were run in a 1.5% agarose gel and visualized in a Molecular Imager GelDoc (Bio-Rad) to confirm their size and specificity of PCR reaction.

### Quantitative real time PCR (qRT-PCR)

Quantitative PCR was performed in an iQ5 thermocycler (Bio-Rad) using 96-well microtitre plates and the QuantiTect SYBR Green PCR Master Mix (QIAGEN) as described before [24]. The cDNA was diluted 100× times in RNase-free water. The primers for the target genes (rat NPY, AgRP, POMC, CART and BDNF) and the reference gene (rat HPRT) were pre-designed and validated by QIAGEN (QuantiTect Primers, QIAGEN). A master mix was prepared for each primer set containing the appropriate volume of 2× QuantiTect SYBR Green PCR Master Mix and 10× QuantiTect Primer (both from QIAGEN). For each reaction, 18 µl of master mix were added to 2 µl of template cDNA. All reactions were performed in duplicate (two cDNA reactions per RNA sample). The reactions were performed according to the manufacturer's recommendations: 95°C for 15 min. followed by 40 cycles of 94°C for 15 sec, 55°C for 30 sec and 72°C for 30 sec. The melting curve protocol started immediately after amplification. Additionally, the PCR products were run in a 2% agarose gel to confirm their size. PCR runs with RNA samples (no RT samples) served as negative controls. The amplification efficiency for each gene and the threshold values for threshold cycle determination (Ct) were determined automatically by the iQ5 Optical System Software (Bio-Rad).

Relative mRNA quantification was performed using the ΔCt method for genes with the same amplification efficiency. The mRNA quantification of different neuropeptides was performed in the same culture preparations. For the experiments using inhibitors the mRNA quantification is shown as percentage of control. The procedure was performed for five independent culture preparations.

### Statistical analysis

All the experiments were replicated in four to five independent culture preparations. The values in the figures are expressed as mean ± SEM. For the experiments involving fluoxetine and inhibitors treatment, data were analyzed by one-way ANOVA with post hoc test to determine significant differences between groups. For the experiments involving neuropeptides quantification during passages and neurospheres percentage for size intervals, data were analyzed by two-way ANOVA with post hoc test to determine significant differences between control and fluoxetine groups.

## Results

### Hypothalamic neuroprogenitor cells and differentiated hypothalamic neuroprogenitor cells express serotonergic markers

**Figure 1:**
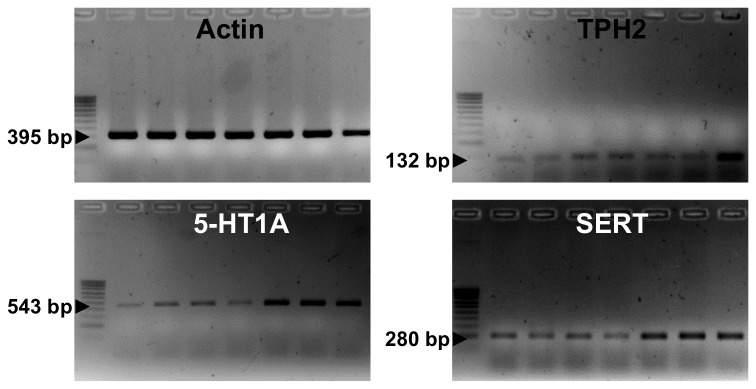
Presence of serotonergic markers in hypothalamic neuroprogenitor cells. Representative gel electrophoresis with PCR products demonstrating the presence of β-actin (**A**), neuronal tryptophan hidroxylase (TPH2) (**B**), serotonin receptor 5-HT_1_A (**C**) and serotonin pre-synaptic transport (SERT) (**D**) in hypothalamic neuroprogenitor cells. PCR was performed in cDNA samples obtained from hypothalamic neurospheres: (1) - P1 control; (2) P1 treated with fluoxetine; (3) - P3 control; (4) - P3 treated with fluoxetine; (5) – differentiated control; (6) – differentiated treated with fluoxetine; and (+) whole adult brain (positive control). P, passage.

Before investigating the putative actions of the selective serotonin reuptake inhibitor fluoxetine in the proliferation/maintenance of hypothalamic neuroprogenitor cells, we evaluated the presence of serotonergic markers in the cultures (Figure 1). PCR revealed the presence of mRNAs for serotonin synthesis enzyme neuronal tryptophan hydroxylase (TPH2) (Figure 1-B), neurogenesis associated serotonin receptor 5-HT_1_A (Figure 1-C) and serotonin pre-synaptic transporter (SERT) (Figure 1-D) in hypothalamic neuroprogenitor cells cultured as neurospheres and after differentiation.

### Fluoxetine promotes the proliferation of hypothalamic neuroprogenitor cells

In order to evaluate the neurogenic effects of fluoxetine in hypothalamic neuroprogenitor cells, we mimic the perinatal exposure to this drug by incubating hypothalamic neurospheres with fluoxetine for up to 5 weeks. In rodents, hypothalamic neuronal precursors can be isolated from the early embryonic period until adulthood [5], [6], [7]. Therefore, we obtained the hypothalamic neurospheres from E18-19 rat embryos as previously described by our group [8]. The incubation period was based on previous reports showing that maximal increase in neurogenesis achieved by fluoxetine and other SSRIs occurs after a prolonged treatment, in adult mice hippocampus [27], [28].

**Figure 2:**
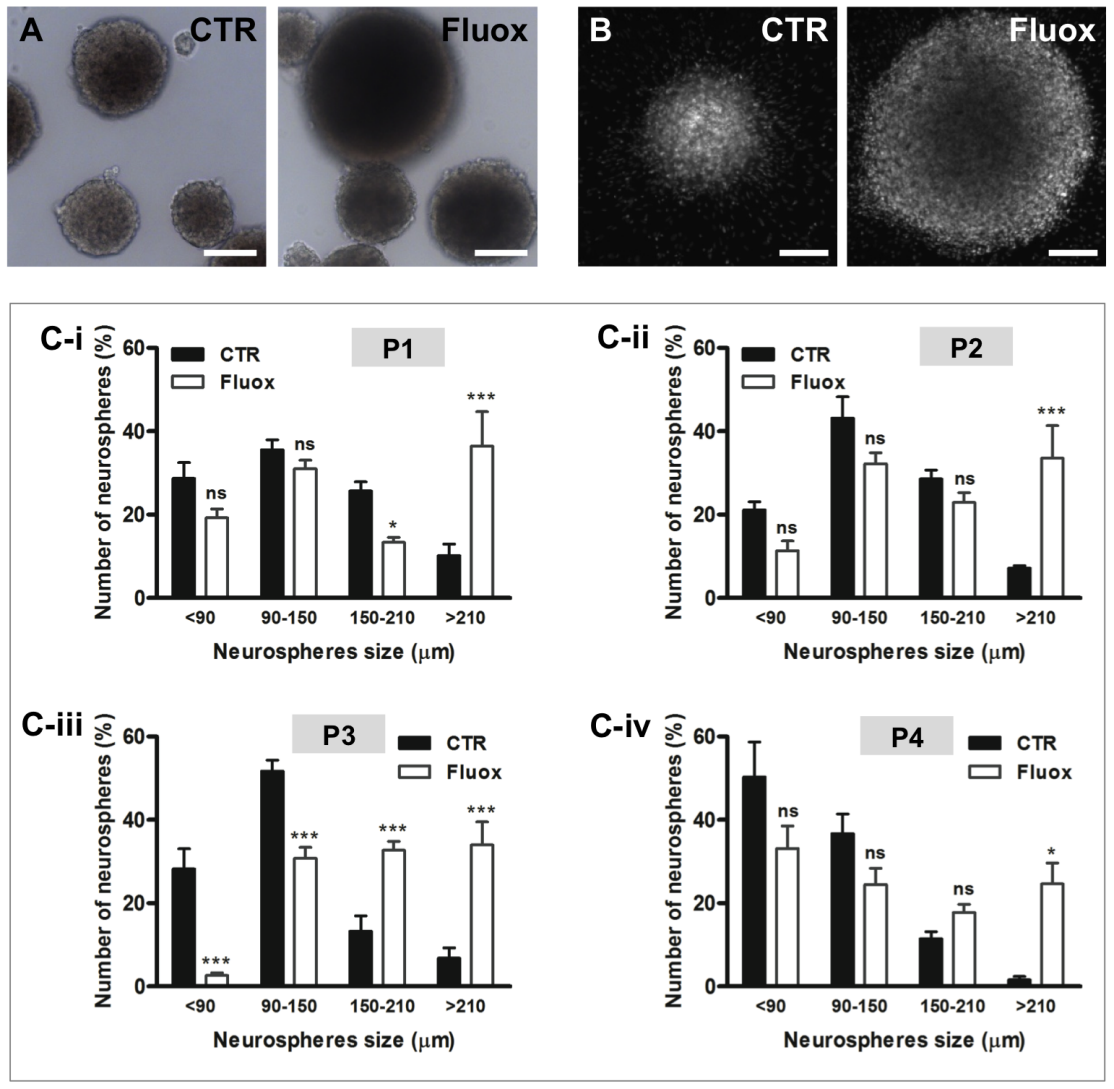
Fluoxetine increases the size of hypothalamic neurospheres. Hypothalamic neurospheres were incubated with fluoxetine (1 µM) throughout 3 passages. Representative images of P3 hypothalamic neurospheres morphology showed by phase-contrast microscopy (**A**), and nuclear staining with DAPI (white) (**B**). (**C**) Diameter distribution of hypothalamic neurospheres in passages 1 to 4. Mean ± SEM; n = 3/4; Two-way ANOVA; ns, p>0.05; *, p<0.05; **, p<0.01, ***, p<0.001 compared to control. P, passage. Scale bar: 100 µm.

We first observed that, morphologically, neurospheres exposed to fluoxetine were larger than control neurospheres, as shown in phase-contrast microscopy (Figure 2-A) and nuclear staining with DAPI (Figure 2-B). To further evaluate this effect, we measured the diameter of hypothalamic neurospheres throughout the passages and compared the size distribution (Figure 2-C).

Fluoxetine drastically increased the percentage of >210 µm diameter neurospheres at the passages P1, P2 and P4 (Figure 2-C). Additionally, fluoxetine showed the strongest effect at the third passage (P3) when it induced an increase in the percentage of large neurospheres (150–210 µm and >210 µm diameter neurospheres), together with a decrease in the percentage of small neurospheres (<90 µm and 90–150 µm diameter neurospheres) (Figure 2-Ciii). These data demonstrating an increase in the size of neurospheres indicate that fluoxetine promotes the proliferation and/or survival of hypothalamic neuroprogenitor cells.

**Figure 3:**
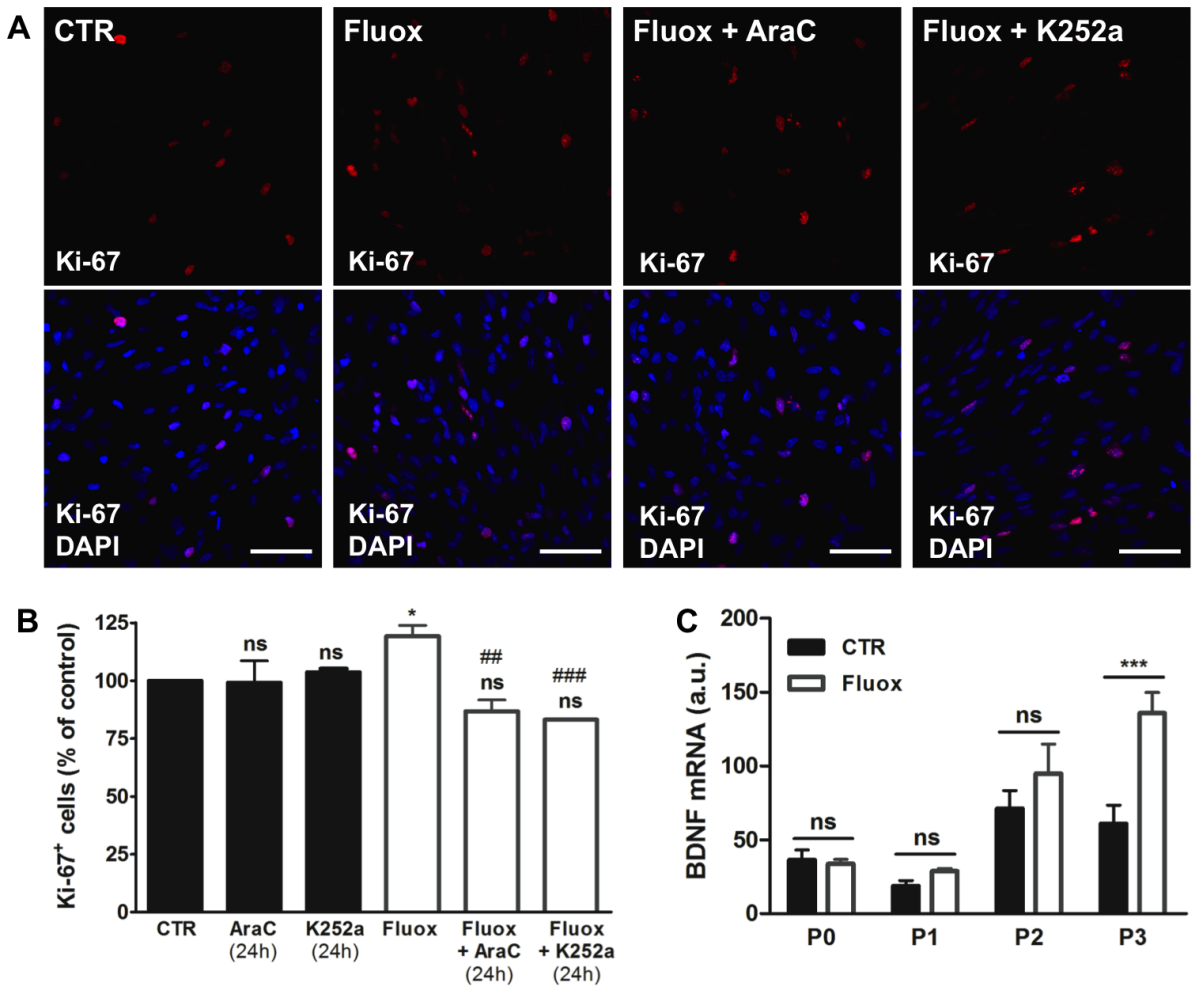
Fluoxetine promotes the proliferation of hypothalamic neuroprogenitor cells. Hypothalamic neurospheres were incubated with fluoxetine (1 µM) throughout 3 passages. Representative confocal photomicrographs of cell proliferation marker Ki-67 (red) (**A**). Fluoxetine increases the percentage of Ki-67 positive cells and 24 hours incubation with proliferation inhibitor AraC or Trk receptors inhibitor K252a reverses this effect (**B**). Fluoxetine upregulates the mRNA levels of neurotrophic factor BDNF (**C**). DAPI (blue), nuclear staining. Mean ± SEM; n = 4; One-Way ANOVA (B) and Two-Way ANOVA (C); ns, p>0.05; *, p<0.05; ***, p<0.001 compared to control; ^##^, p<0.01; ^###^, p<0.001 compared to fluoxetine. Scale bar: 50 µm. P, passage.

To clarify this effect, we evaluated the number of Ki-67 positive cells; Ki-67 is a commonly used proliferation marker that stains cells in active cell cycle [4]. In P3 neurospheres (the passage when the effect of fluoxetine on neurospheres diameter was more evident) we observed that fluoxetine increased the immunostaining for Ki-67 (Figure 3-A), with a 20% increase in the percentage of Ki-67 positive cells compared to control (Figure 3-B).

### Involvement of BDNF in the proliferation of hypothalamic neuroprogenitor cells promoted by fluoxetine

It has been reported that the activation of hippocampal neurogenesis induced by antidepressants involves an upregulation of BDNF (Brain-Derived Neurotrophic Factor) [29], [30]. BDNF is a neurotrophin expressed in the adult rodent hypothalamus [31] that promotes neurogenesis in this brain region after infusion to the third ventricle [3]. Interestingly, in our cell model, we observed that fluoxetine significantly upregulated the levels of BDNF in P3 hypothalamic neurospheres, but not in earlier passages, with a 2-fold increase in the BDNF mRNA when compared to control, as shown in Figure 3-C.

The neurobiological actions of neurotrophins are mediated by a family a tyrosine kinase receptors: TrkA, TrkB and TrkC receptors. Therefore, to investigate the involvement of neurotrophins in the proliferation of hypothalamic neuroprogenitor cells induced by fluoxetine we used K252a, a Trk receptors inhibitor. K252a is an alkaloid extensively used as Trk receptors antagonist, [29], [32], [33], [34], which can block the different types of Trk receptors and prevent the action of neurotrophins that exist in the hypothalamic neurospheres cultures.

In our study, we quantified the number of Ki-67 positive cells after incubation with cell proliferation inhibitor AraC or Trk receptors inhibitor K252a, during the last 24 hours before cell fixation. The elevated number of proliferative cells was reversed to control levels by incubation with AraC or K252a in fluoxetine treated cultures (Figure 3-A and B). Both inhibitors had no effect in the number of proliferative cells in control neurospheres (Figure 3-B). Since Ki-67 reveals cells in active cycle at the time of fixation, these results show that the 24 hours incubation with inhibitors was sufficient to block the fluoxetine-induced proliferation. Moreover, the fact that 24 hours incubation with AraC is sufficient to decrease fluoxetine-induced, but not basal, proliferation suggests that cells growing in the presence of this antidepressant drug present higher proliferation rates and incorporate proliferation inhibitor AraC faster than control cells.

Additionally, incubation of P3 neurospheres with AraC did not change significantly the expression of BDNF mRNA in both conditions (Figure S1). However, the incubation with K252a, induced a compensatory increase in BNDF mRNA both in control and fluoxetine P3 neurospheres (Figure S1), suggesting a compensatory increase of BDNF expression upon the blockade of Trk receptors.

Overall, these results show that fluoxetine promotes the proliferation of hypothalamic neuroprogenitor cells, via a mechanism involving neurotrophins.

### Fluoxetine upregulates the levels of orexigenic neuropeptides, but not of anorexigenic neuropeptides, in hypothalamic neurospheres

**Figure 4:**
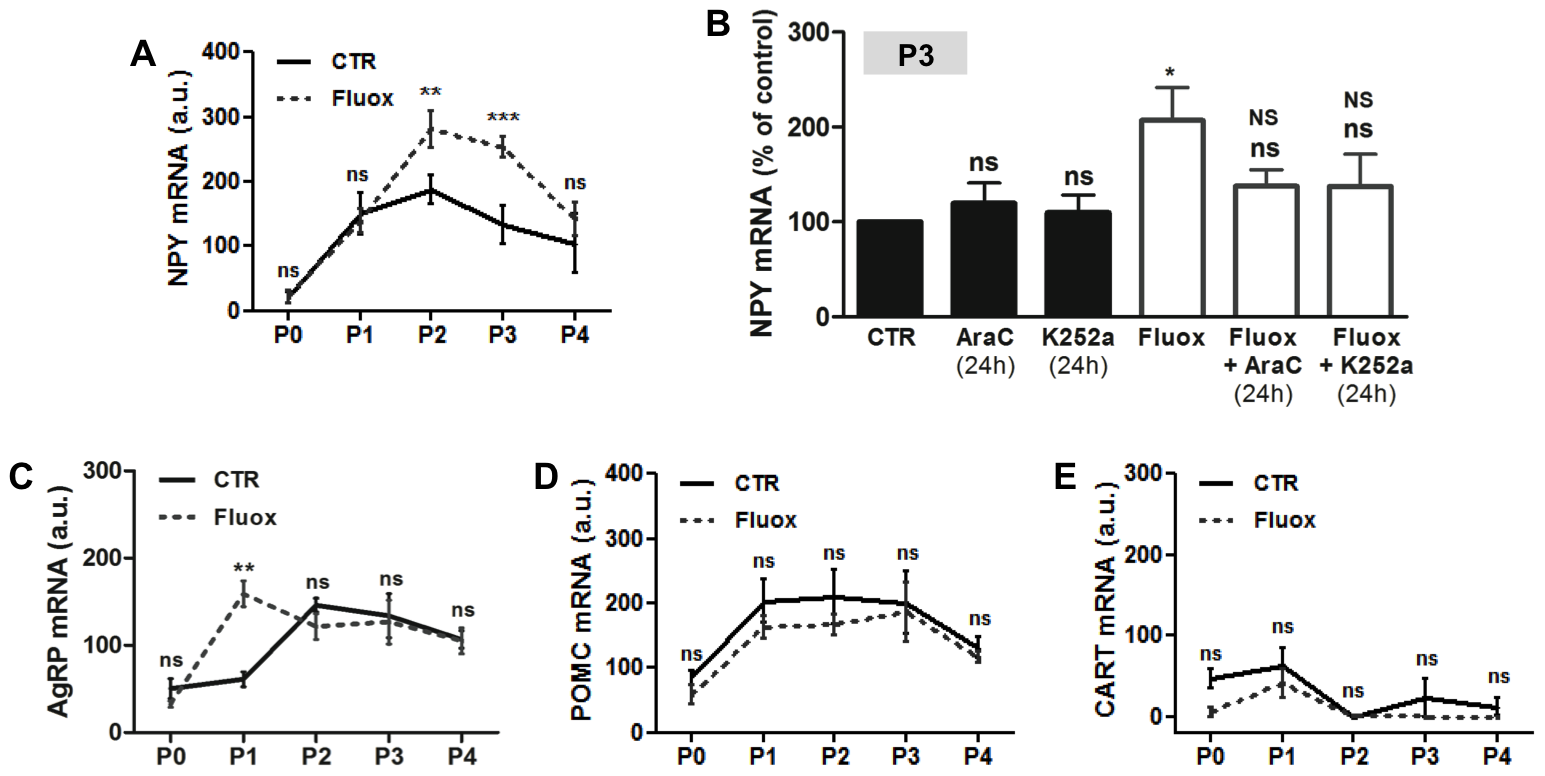
Fluoxetine upregulates the levels of orexigenic neuropeptides NPY and AgRP in hypothalamic neuroprogenitor cells. Fluoxetine increases the mRNA levels of NPY during P2 and P3 (**A**). The 24 hours incubation with proliferation inhibitor AraC and Trk receptor inhibitor K252a reverses the increase of NPY mRNA induced by fluoxetine (**B**). Fluoxetine anticipates the increase of AgRP mRNA in P1 neurospheres (**C**). Fluoxetine has no effect in the mRNA levels of the anorexigenic neuropeptides: POMC (**D**) and CART (**E**). Mean ± SEM; n = 4; Two-Way ANOVA (A, C, D, E) and One-Way ANOVA (B); ns, p>0.05; *, p<0.05; **, p<0.01; ***, p<0.001 compared to control; NS, p>0.05 compared to fluoxetine. P, passage.

Proliferation changes during hypothalamic neurodevelopment can lead to an impaired balance between neurons expressing orexigenic and anorexigenic neuropeptides [10], [11]. Since hypothalamic neuroprogenitor cells obtained from rat embryos express neuropeptides important for the regulation of food intake [8], we used the neurospheres cell model to evaluate the effect of fluoxetine in the mRNA expression of orexigenic (NPY and AgRP) and anorexigenic (POMC and CART) neuropeptides (Figure 4).

Concerning the orexigenic neuropeptides, fluoxetine significantly increased the expression of NPY in hypothalamic neurospheres, with a 1.5-fold increase in mRNA levels at P2, and a 2-fold increase at P3 (Figure 4-A). To investigate whether the upregulation of NPY was dependent of the proliferative effect of fluoxetine in hypothalamic neuroprogenitor cells, we incubated P3 neurospheres with proliferation inhibitor AraC (24 h) and Trk receptors inhibitor K252a (24 h). In fact, the NPY mRNA returned to control levels with AraC or K252a incubation (Figure 4-B), indicating that both proliferation and Trk receptors mediate the upregulation of NPY levels induced by fluoxetine in hypothalamic neuroprogenitor cells. Both inhibitors had no effect in the NPY mRNA levels in control neurospheres (Figure 4-B).

Additionally, fluoxetine led to an earlier rise on AgRP levels, with a 5-fold increase in the mRNA levels occurring at P1 (Figure 4-C). In control neurospheres, the 2.5-fold increase in the expression of this neuropeptide occurred later at P2 (Figure 4-C). In opposition, the levels of anorexigenic neuropeptides were not different between control and fluoxetine treated neurospheres along the passages, as shown by mRNA levels of POMC (Figure 4-D) and CART (Figure 4-E).

All together, these data show that fluoxetine modifies the expression levels of neuropeptides important for the regulation of feeding and that this effect is dependent of the proliferative effect of fluoxetine on hypothalamic neuroprogenitor cells.

### Fluoxetine inhibits the differentiation of hypothalamic neuroprogenitor cells

**Figure 5:**
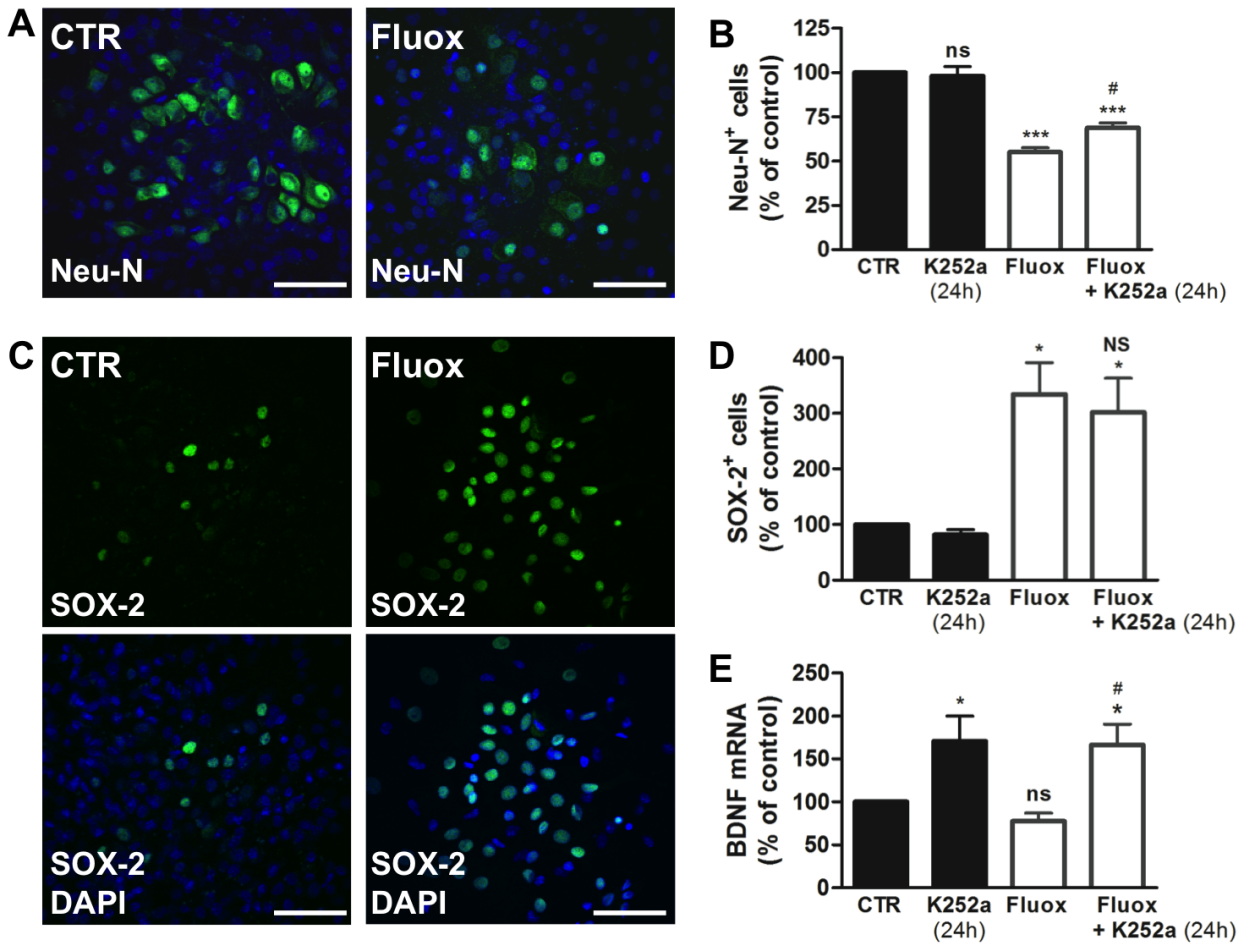
Fluoxetine inhibits the differentiation of hypothalamic neuroprogenitor cells. Hypothalamic neuroprogenitor cells were differentiated for 18 days, in the presence or absence of fluoxetine (1 µM). Representative confocal photomicrographs for mature neurons marker Neu-N (green) (**A**). Fluoxetine decreases the percentage of Neu-N positive cells and the incubation with Trk receptors inhibitor K252a (24 h) partially reverses this effect (**B**). Representative confocal photomicrographs for the progenitor cells marker SOX-2 (green) (**C**). Fluoxetine increases the percentage of SOX-2 positive cells and the incubation with K252a (24 h) does not change this effect (**D**). Fluoxetine does not up regulate the levels of neurotrophic factor BDNF but incubation with K252a (24 h) results in a compensatory increase of BDNF mRNA levels (**E**). DAPI (blue), nuclear staining. Mean ± SEM; n = 4/5; One-Way ANOVA; ns, p>0.05; *, p<0.05; ***, p<0.001 compared to control; NS, p>0.05, ^#^, p<0.05 compared to fluoxetine. Scale bar: 50 µm.

To evaluate the effects of fluoxetine during the differentiation of hypothalamic neuroprogenitor cells, we allowed the neurospheres to differentiate for 18 days in presence or absence of fluoxetine. After that, the differentiation profile was evaluated by quantification of the number of mature neurons (Neu-N^+^ cells) and progenitor cells (SOX-2^+^ cells) [35]. Hypothalamic neuroprogenitor cells differentiated in the presence of fluoxetine showed a decreased immunostaining for mature neurons marker (Figure 5-A) with a 45±2.5% reduction in the percentage of Neu-N positive cells, compared to untreated cells (Figure 5-B). Moreover, the number of progenitor cells (SOX-2^+^ cells) was robustly increased in the cultures incubated with fluoxetine during differentiation (Figure 5-C), with a 3.3-fold increase in the percentage of SOX-2 positive cells compared to control conditions (Figure 5-D). Together these evidences suggest that fluoxetine inhibits the neuronal differentiation of hypothalamic neuroprogenitor cells and maintains their undifferentiated status.

To evaluate the contribution of neurotrophins to the maintenance of hypothalamic neuroprogenitor cells in the undifferentiated state we first evaluated the expression of BDNF (Figure 5-E). The mRNA levels of BDNF were not changed in the cultures differentiated in the presence of fluoxetine as compared to control cultures (Figure 5-E). However, there was a compensatory increase in the mRNA levels of BDNF after incubation with Trk receptors inhibitor K252a for 24 hours (Figure 5-E), similar to the one observed for the hypothalamic neurospheres.

On the other hand, the effect of fluoxetine on reducing the number of Neu-N positive cells was partially inhibited by incubation with Trk receptor inhibitor K252a for 24 hours (Figure 5-B). Nevertheless, this inhibitor did not change the number of SOX-2 positive cells (Figure 5-D).

### Fluoxetine decreases the levels of abundant neuropeptides in differentiated hypothalamic neuroprogenitor cells

Differentiated hypothalamic neuroprogenitor cells express neuropeptides important for the regulation of food intake, including NPY, AgRP, POMC and CART [8]. To investigate whether fluoxetine affects the expression of these neuropeptides, we quantified their mRNA levels after differentiation in the presence or absence of fluoxetine.

**Figure 6:**
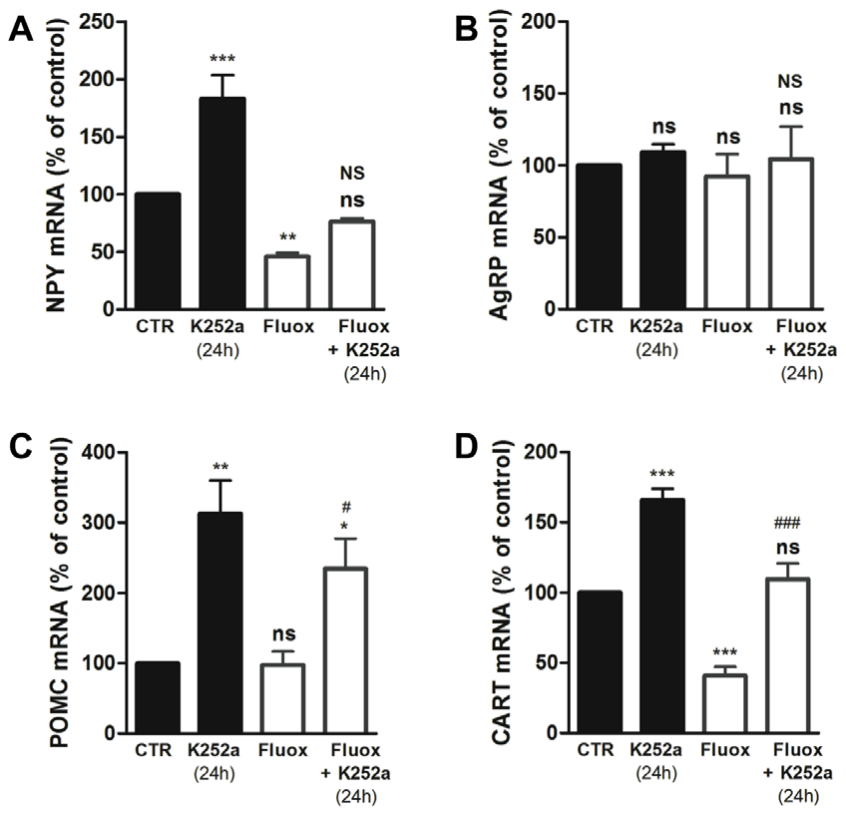
Fluoxetine decreases the levels of abundant neuropeptides (NPY and CART) in hypothalamic differentiated neuroprogenitor cells. Fluoxetine decreases the mRNA levels of neuropeptides that are abundant in differentiated hypothalamic neuroprogenitor cultures NPY (**A**) and CART (**D**), but has no effect in the mRNA of AgRP (**B**) and POMC (**C**). Mean ± SEM; n = 5; One-Way ANOVA; ns, p>0.05; *, p<0.05; **, p<0.01; ***, p<0.001 compared to control; NS, p>0.05, ^#^, p<0.05, ^###^, p<0.001 compared to fluoxetine.

Hypothalamic neuroprogenitor cells differentiated in the presence of fluoxetine showed a 54.0±3.3% decrease in the NPY mRNA levels (Figure 6-A) and a 59.0±6.3% decrease in the CART mRNA levels (Figure 6-D). In opposition, the mRNA levels of AgRP (Figure 6-B) and POMC (Figure 6-C) were not affected.

Interestingly, NPY and CART are the neuropeptides that showed a higher enrichment during the neuronal differentiation of hypothalamic neuroprogenitor cells, as previously reported by our group [8]. Therefore, we can hypothesize that fluoxetine, by decreasing the neuronal differentiation of these cells, inhibits the elevation of abundant neuropeptides NPY and CART mRNA but has no effect on the levels of less abundant neuropeptides AgRP and POMC.

Additionally, hypothalamic neuroprogenitor cells were incubated with Trk receptors inhibitor K252a for 24 hours, to evaluate the involvement of Trk receptors in the differentiation of these cells. Incubation with K252a led to a wide increase in the levels of neuropeptides NPY, POMC and CART, but not AgRP, that occurred independently of fluoxetine treatment (Figure 6). Therefore, the effect of K252a in neuropeptides levels does not seem to be related to the blockade of Trk receptors. Other properties of K252a, such as stabilization of calcium homeostasis and support of neuronal survivor [36], may be associated to this increase of neuropeptides levels.

## Discussion

The present work shows that the antidepressant drug fluoxetine promotes the proliferation and maintenance of hypothalamic neuroprogenitor cells and, by means of this effect, changes the expression of neuropeptides important for the regulation of feeding. Understanding the actions of SSRIs in the embryonic hypothalamus is of utmost importance since antidepressants therapy is frequent among women in the perinatal period [15] and energy balance alterations in newborns have been described after maternal exposure to antidepressants [20], [21], [22], [24].

Notably, we demonstrate that hypothalamic neuroprogenitor cells are susceptible to SSRI exposure as shown by the presence of serotonergic markers, including serotonin synthesis enzyme (TPH2), pre-synaptic transporter and 5-HT_1_A receptor. Accordingly, the 5-HT_1_A receptor was previously associated to the neurogenic effect of antidepressants in the hippocampus [27], [37].

### Fluoxetine promotes the proliferation and maintenance of hypothalamic neuroprogenitor cells

In this study we have investigated the neurogenic actions of fluoxetine in hypothalamic neuroprogenitor cells cultured as neurospheres. This model presents the limitations of an *in vitro* model, particularly the possibility that the mechanisms observed for the cell culture preparations will not be recapitulated in the living organism. Nevertheless, the neurospheres culture model has been extensively used by researchers for studying proliferation of well-known neurogenic regions like the lateral ventricles and hippocampus [38], [39]. More recently, this model has been used for investigating neurogenesis in the embryonic hypothalamus [9], [10] and adult hypothalamus [6], [40], [41]. Additionally, this is an interesting model to study hypothalamic neurogenesis since proliferation mechanisms described for hypothalamic neurospheres cultures are often recapitulated in the rodent hypothalamus [6], [40], [41].

Fluoxetine promotes the proliferation and survival of new neurons in the adult hippocampus [27], [28] by increasing the symmetric divisions of early progenitor cell [42]. However, the possible neurogenic effect of this SSRI in the hypothalamus was unknown. With this work, we demonstrate that fluoxetine stimulates the proliferation of neuroprogenitor cells obtained from the fetal hypothalamus, and maintains the hypothalamic neuroprogenitor cells in the undifferentiated state. Additionally, we showed that the proliferative effect of fluoxetine in hypothalamic neuroprogenitor cells is mediated by neurotrophins.

Furthermore, the involvement of BDNF is suggested by the data showing an upregulation of BDNF mRNA levels in the neurospheres treated with fluoxetine at P3, the passage with maximal proliferative effect (as shown with neurospheres size). Noteworthy, previous reports showing that the response of neuroprogenitor cells to fluoxetine is mediated by an upregulation of BDNF levels in the hippocampus [29], [30], [43] and that a chronic administration of fluoxetine is needed to initiate the proliferation of new neurons in the hippocampus of mice [27], [28], support the role of BDNF as mediator of fluoxetine neurogenic actions.

Moreover, BDNF is an important molecule to the cellular physiology of the hypothalamus since it is involved in the neuronal development and maturation of this brain region [44], [45] and in the stimulation hypothalamic neurogenesis [3]. Considering these evidences, it is even possible that BDNF plays a role in the basal maintenance of hypothalamic neuroprogenitor cells (although this hypothesis was not investigated in the present study).

In opposition, the inhibition of neuronal differentiation produced by fluoxetine does not seem to be mediated by neurotrophins: the mRNA level of BDNF is similar in hypothalamic neuroprogenitor cells differentiated in the presence or absence of fluoxetine and Trk receptors inhibitor does not reverse the high number of SOX-2 positive cells observed in fluoxetine-treated cell cultures. However, the incubation with K252a partially reversed the low number of mature neurons quantified in cultures differentiated in the presence of this antidepressant. Other mediators of fluoxetine neurogenic activity, such as the recently described glial cell-derived neurotrophic factor (GDNF), cyclin-dependent kinase inhibitor p21 and Wnt3a [29], [46], [47], may be responsible for the effect observed in our study but their evaluation was beyond the scope of this article.

### Fluoxetine modifies the expression of neuropeptides that regulate food intake

In this study, we showed that fluoxetine alters the expression levels of neuropeptides important for the regulation of appetite, including upregulation of orexigenic neuropeptides NPY and AgRP, during the proliferation of hypothalamic neuroprogenitor cells, and down regulation of abundant neuropeptides NPY and CART, during the differentiation of these cells. Moreover, our results suggest that the alterations of neuropeptides levels promoted by fluoxetine are mediated by its effects in the proliferation and maintenance of neuroprogenitor cells.

The expression of NPY (and other neuropeptides) in embryonic mitotic cells, and in particular, in hypothalamic neuroprogenitor/neuroprecursor cells was previously demonstrated in neurospheres cultures [8] and mice hypothalamus [5], [6]. However, the presence of specific neuropeptides in these cells may not reflect the acquisition of their terminal peptidergic phenotype [5], as this dynamic process occurring during the prenatal development of feeding circuits is not concluded until the postnatal period [48], [49]. Nevertheless, it raises the possibility that factors influencing cell fate decisions within this period can permanently affect the hypothalamic peptidergic composition.

Therefore, changes of proliferation and neuropeptides content in hypothalamic neuroprogenitor cells induced by fluoxetine may influence the neuronal differentiation process. This hypothesis is in accordance to a previous study showing that the neurogenesis of orexigenic precursors can be altered by stimuli reaching the hypothalamus and lead to increased density of orexigenic neurons [11].

The involvement of neurotrophins in the effects of fluoxetine further supports the possibility that fluoxetine modifies the expression of neuropeptides by promoting the propagation of hypothalamic neuroprogenitor cells. First, the most significant increase of NPY mRNA occurs simultaneously with the BDNF upregulation and the most robust increase in cellular proliferation. Second, the levels of NPY mRNA return to control after incubation with Trk receptors inhibitor, as it occurred with proliferation inhibitor. Similarly to the data reported here, BDNF upregulates the levels of NPY in cortical cell cultures and hippocampal slices [50], [51], as well as in the hippocampus and cortex after continuous infusion [52], [53].

Surprisingly, previous studies reporting the effects of fluoxetine and BDNF in the adult hypothalamus show opposite evidences from the ones reported in this work. For example, administration of fluoxetine and other serotonergic drugs decreases the content and release of NPY [54], [55], [56] and does not upregulate the levels of BDNF [57] in the hypothalamus of adult rats.

These evidences lead us to hypothesize that the SSRI fluoxetine has divergent actions in mature and neuroprogenitor hypothalamic cells, with the neurogenic activity being more evident on this last type of cells (and responsible for the alterations of neuropeptides levels observed in this study). Our hypothesis is further supported by previous reports showing that the cognitive outcomes of serotonin transporter blockade during development are sometimes dramatically different from the effects during adulthood [58].

Moreover, we can speculate that due to the reduced number of neuroprogenitor cells in the adult hypothalamus [3], the effects of serotonergic analogs in neuroprogenitor cells are more clearly observed in the *in vitro* model described here. This hypothesis reinforces the need of understanding the consequences of exposing embryonic and adult hypothalamic neuroprogenitor cells to antidepressants. In this context, the *in vitro* model used for the present study can represent a valuable model to screen possible neurogenic effects of other drugs in hypothalamic neuroprogenitor cells.

### Conclusion

The importance of adult hypothalamic neurogenesis and plasticity to the metabolic response to dietary challenges was recently demonstrated [4], [6]. But the maternal exposure to molecules that modify hypothalamic proliferation in the prenatal period may compromise that neurogenic capacity in adulthood, as demonstrated for diet lipids and hormones [10], [11].

Our *in vitro* study shows that SSRIs antidepressants have the potential to change the proliferation/differentiation of neuroprogenitor cells from the embryonic hypothalamus. Although future *in vivo* studies are needed, based on the presented results, we can speculate that antidepressant drugs may be yet another contributor to impaired neurodevelopment of this key metabolism center by increasing the pool of neuroprogenitor cells and inhibiting the differentiation of these cells.

## Supporting Information

Figure S1 **Trk receptors inhibitor K252a upregulates the levels of BDNF in P3 hypothalamic neurospheres.** Incubation with K252a for 24 hours results in a compensatory upregulation of the mRNA levels of neurotrophic factor BDNF. Incubation with proliferation inhibitor AraC for 24 hours does not modify the mRNA of BDNF. One-Way ANOVA; ns, p>0.05; *, p<0.05 compared to control. P, passage.
(TIFF)
