# Supplementary figures and images for: Fluoxetine Induces Proliferation and Inhibits Differentiation of Hypothalamic Neuroprogenitor Cells In Vitro
Source: PLoS One. 2014 Mar 5;9(3):e88917. doi: 10.1371/journal.pone.0088917 (PMC3943792; doi:10.1371/journal.pone.0088917)

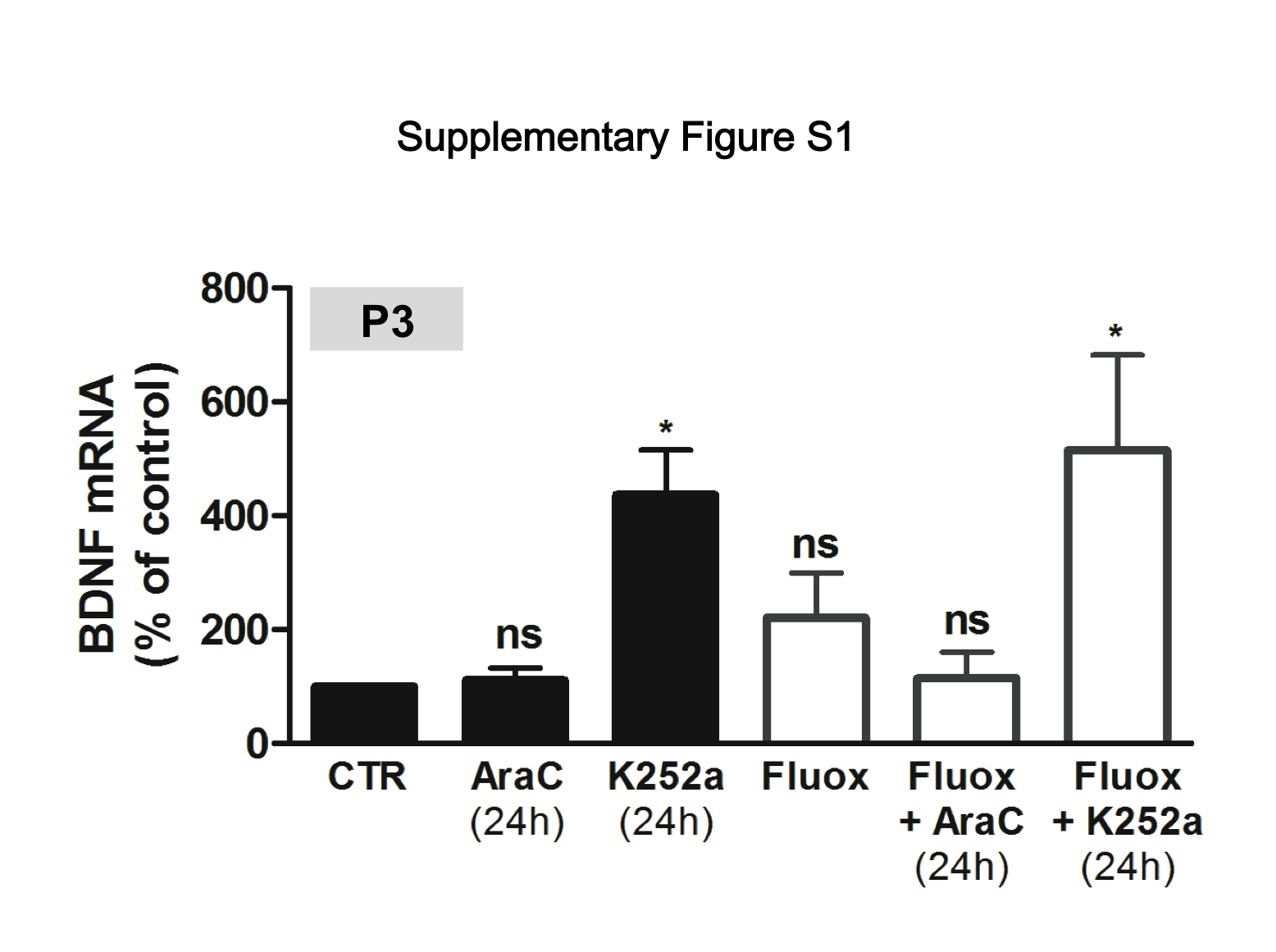

Supplement: Figure S1 — Trk receptors inhibitor K252a upregulates the levels of BDNF in P3 hypothalamic neurospheres. Incubation with K252a for 24 hours results in a compensatory upregulation of the mRNA levels of neurotrophic factor BDNF. Incubation with proliferation inhibitor AraC for 24 hours does not modify the mRNA of BDNF. One-Way ANOVA; ns, p>0.05; *, p<0.05 compared to control. P, passage. (TIFF) [file pone.0088917.s001.tif]
